# The effect of the pooling method on the live birth rate in poor ovarian responders according to the Bologna criteria

**DOI:** 10.4274/tjod.62447

**Published:** 2018-03-29

**Authors:** Serdar Çelik, Niyazi Emre Turgut, Dilek Cengiz Çelik, Kübra Boynukalın, Remzi Abalı, Sevim Purisa, Erbil Yağmur, Mustafa Bahçeci

**Affiliations:** 1 Bahçeci Fulya In Vitro Fertilization Center, Clinic of Obstetrics and Gynecology, İstanbul, Turkey; 2 İstanbul University Faculty of Medicine, Department of Biostatistics and Medical Informatics, İstanbul, Turkey

**Keywords:** Poor responder, frozen embryo transfer, in vitro fertilization

## Abstract

**Objective:**

Pooling is an alternative method to achieve in vitro fertilization outcomes. This study was to investigate the effect of pooling method on pregnancy outcomes in poor responder patients according to Bologna criteria.

**Materials and Methods:**

Two hundred-fifty five poor responder patients were enrolled in this study. Pooling embryo transfer (ET) group had 110 and fresh ET group had 145 patients.

**Results:**

Although, age was similar between both treatment groups (p=0.31), antral follicle count (p<0.001), total number of retrieved oocyte (p<0.001), total metaphase II oocyte count (p<0.001), number of stimulation cycles (p<0.001), were significantly different between the groups. The day of ET were similiar between two groups (p=0.72) but the number of ET procedure was significantly higher in pooling ET group compared to fresh ET (p<0.001). Positive pregnancy test [35/110 (32%) vs 53/145 (37%)] (p=0.43) and clinical pregnacy rates [31/110 (28%) vs 49/145 (34%)] (p=0.33) were similar between groups, whereas, implantation [31/191 (16%) vs 49/198 (25%)] (p=0.03) and live birth rates [15/110 (14%) vs 36/145 (25%)] (p=0.04) were significantly higher in fresh ET group. Despite that, abortion rates were significantly higher in pooling ET group [16/31 (52%) vs 13/49 (27%)] (p=0.04). Binary logistic regression analyese has revealed no effect of variables on live birth rates.

**Conclusion:**

Even though, pooling strategy seems to have a slight positive effect on pregnancy outcomes, there is no benefical effect on live birth rates. Furthermore, this strategy is increasing the abortion rates in parallel with clinical pregnancy rates.

**PRECIS:** Pooling does not increase live birth rates among poor responder patients.

## Introduction

There is ongoing debate about the management of poor responder women in *in vitro* fertilisation (IVF) centres. Although they receive an increased gonadotropin dose compared with normoresponders, fewer oocytes are eligible for the procedure, and thus, the pregnancy outcomes are lower^(1,2,3)^. Therefore, physicians have focussed on other methods of increasing pregnancy rates in poor responder women. Several treatment options, such as oestrogen use in the luteal phase^(4)^, adding a recombinant luteinizing hormone preparation during stimulation^(5)^, and pre-treatment with growth hormone^(6)^ and androgen^(1)^ have been investigated. Yet, lower pregnancy rates are still reported in poor responders compared with normoresponder women. In recent years, the cryopreservation of embryos has become an essential component of treatment with assisted reproductive technology, and due to the technological developments in the embryology arm, frozen/thawed embryo transfer (FET) has been offered as an alternative to physicians. Two methods are commonly used-slow freezing and vitrification. Recently, Sites et al.^(7)^ reported significantly lower live birth rates with slow freezing compared with vitrification (25% vs 71%) and fresh embryo transfer (ET) (ET; 25% vs 70%)^(8)^. FET has become an alternative method to fresh ET in normoresponder women, but there is no consensus about the use of FET in poor responder patients. The number of retrieved oocytes is correlated with the birth rate^(9)^. Management options are scarce in poor responder women because of the lower oocyte numbers and suboptimal oocyte maturation^(10)^. Increasing the embryo yield via an accumulation from consecutive stimulation cycles may be a new approach to overcome poor outcomes. Accumulated embryos from consecutive stimulation cycles are frozen and hidden by vitrification, and ET is performed by thawing the entire cohort after reaching the proper number. Theoretically, similar pregnancy and delivery rates to those of normoresponder patients may be achieved. There are many different definitions of poor responders in the literature^(11,12,13)^. Most recently, poor responders were defined as detailed by Ferraretti et al.^(14)^. We consider that sufficient pregnancies can be achieved if enough oocytes are retrieved in consecutive cycles. Thus, the aim of this study was to investigate the effect of embryo collection on the pregnancy, clinical pregnancy and live birth rates in poor responder women, as defined according to the Bologna criteria^(14)^.

## Materials and Methods

The study was performed at the Bahçeci Fulya IVF Centre. All patients who underwent ET procedures were screened using electronic records from August 2010 to January 2014. Ethics committee approval was not required for this study because it involved retrospective data analysis. Nevertheless, a consent form was signed by all participants, and clinical investigation commission approval was received. We declare that we have no financial or personal relationships with other people or organisations that could inappropriately influence our work; there is no professional or other personal interest of any nature or kind related to any product, service and/or company that could be construed as influencing the research. To generate homogeneous study groups and show the power of ET rather than transfer cancellation, we excluded women aged ≥46 years at the time of ET; those who had used neoadjuvant therapy, such as dehydroepiandrosterone and growth hormone, before the procedure; those with controlled ovarian stimulation (COS) regimes other than the letrozole/antagonist protocol for the fresh ET arm; those with notification of difficult ET and use of a different catheter apart from a soft catheter by the performing physician; and those undergoing a second FET from a remaining pool. Patients who met the Bologna criteria as described by Ferraretti et al.^(14)^ were included in the study. To ensure a similar endometrial receptivity between the groups, serum oestradiol (E2) and progesterone (P) levels were determined on the human chorionic gonadotropin (hCG) day of the fresh ET cycle and day 15 of the endometrial preparation cycle in the pooling ET arm. Those with a P level >1.5 ng/mL on the hCG day were excluded from the study. 

Stimulation cycles ending with a preimplantation genetic diagnosis (PGD) for aneuploidy screening or another situation were excluded from both treatment arms. All patients used their own oocytes because egg donation is illegal in Turkey. The live birth rate was considered the primary outcome. The clinical pregnancy and miscarriage rates were considered secondary outcomes. 

### Pooling methods

There was no restriction on the stimulation protocol among the participants recruited for the pooling group. However, for the final oocyte maturation, a fixed 250 µg of recombinant hCG (Ovitrelle, Serono, Turkey) was used subcutaneously for all stimulated cycles. All the embryos were generated by intracytoplasmic sperm injection (ICSI) and vitrified afterwards. At least two COS/ICSI cycles were performed. Embryos recruited from the last cycle were also vitrified. The embryo(s) obtained for each cycle were kept in culture until the blastocyst stage in women undergoing blastocyst ET. Embryo(s) that reached the blastocyst stage were frozen. In women undergoing ET at the cleavage stage, the obtained embryo(s) that reached the cleavage stage were frozen. The whole cohort was thawed on the appropriate day, if vitrified, of the developmental stage and selected for transfer as the best-quality embryos according to the morphologic assessment criteria described below.

### Endometrial preparation

Transdermal E2 hemihydrate patches (Climara Forte, Bayer, İstanbul, Turkey), which were preferred to prime the endometrium, were started on day 3 of menstruation at a dosage of 100 mcg/day for the first 4 days, 200 mcg/day for the next 4 days, and 300 mcg/day for the last 4 days. The serum P level and endometrial thickness were checked afterwards. Vaginal P gel (Crinone 8%, 90 mg, MerckSerono, Bedfordshire, UK) was initiated once per day if the P level was <1.5 ng/mL, endometrial thickness >8 mm, and a triple-line appearance was evident. Transdermal patches at a dosage of 300 mcg/day and vaginal P gel were maintained until the pregnancy test. After a positive test result patients continued to apply the transdermal patches with the same dosage, but the vaginal P was increased to twice per day and continued up to the 10^th^ week of pregnancy. 

### The flexible letrozole/Antagonist protocol

Combination therapy with letrozole (Femara 2.5 mg, Novartis, İstanbul, Turkey) tablets twice per day and 150-450 IU of subcutaneous human menopausal gonadotropin (hMG; Merional, IBSA Institut Biochimique SA, Lamone, Switzerland) injections were started on day 3 of the present cycle. Serial sonographic examinations and serum E2 level measurements were evaluated during the course of follicular development. The gonadotropin-releasing hormone antagonist (Cetrotide, Serono, Turkey) was added at a dosage of 0.25 mg/day when the leading follicle reached 12-14 mm in size and continued up to final triggering. One ampoule of recombinant hCG (Ovitrelle, Serono, Turkey) was administered as soon as the leading follicle reached a mean diameter of 18 mm. Ovum puncture was performed after 36 hours from recombinant hCG injection by transvaginal-ultrasound-guided needle aspiration under general anaesthesia. Cleavage stage embryos or blastocyst transfers were performed afterwards.

### Luteal support

Luteal phase support was initiated on the day of the oocyte pickup procedure for the fresh ET arm and day 15 for the pooling ET arm, as described by Bulent Urman et al.^(15)^.

### The vitrification and embryo thawing protocol

We used our own solutions for embryo vitrification and thawing procedures. The embryos in the cleavage stage were placed for 6-8 minutes and the blastocyst for 10-12 minutes in equilibration solution at room temperature. Afterwards, they were kept in the vitrification solution for 40 seconds just before being transferred into liquid nitrogen. The thawing process was started with the removal of the cyrovials from the liquid nitrogen and keeping the embryos in the first thawing solution at 37 °C for 60 seconds and then in the second solution for 180 seconds at room temperature. Following this, they were transferred into the culture solution to be put in the incubator.

### Embryo and blastocyst morphology

Cleavage-stage embryos were evaluated according to Hardarson et al.^(16)^ description. The morphologic assessment of the blastocysts was performed by means of a staging algorithm, as described by Gardner et al.^(17)^.

### Pregnancy definitions

Serum hCG measurements were evaluated 9 days after blastocyst transfer and 12 days after cleavage stage ET. A value of β hCG >5 mIU/mL was accepted as positive. Clinical pregnancy was defined as an intrauterine sac envisioned by transvaginal sonography at 7 weeks of gestation; the implantation rate was obtained by dividing the number of gestational sacs into the number of transferred embryos^(18)^. Pregnancies that ended before the 24^th^ week of gestation were included in the abortion group. The abortion rate was obtained by dividing the number of pregnancy losses into the number of clinical pregnancies^(19)^. Live birth was defined as the birth of one or more infants with a gestational age of ≥24 weeks^(20)^. Live birth rates per patient and per transferred embryo were calculated separately by dividing the total number of births occurring at a gestational age of ≥24 weeks into the whole cohort and the number of transferred embryos, respectively.

### Statistical Analysis

The distribution of the variables was assessed using a histogram, the Kolmogorov-Smirnov and One Sample tests. In this study, data are presented in terms of median, minimum, maximum, frequency and percentage. The Mann-Whitney U test was used for quantitative variables thought to be effective on live births. The chi-square test was used to compare categorical variables. p values <0.05 were considered statistically significant. Logistic regression analysis was used for variables thought to be effective on live birth outcomes. The analyses were performed using the Statistical Package for the Social Sciences version 21.0. 

## Results

**Figure 1:**
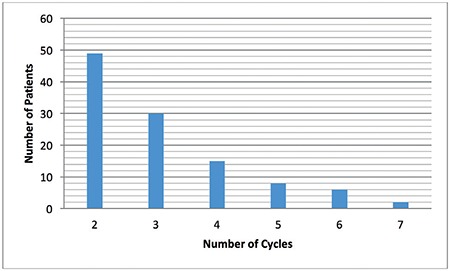
Stimulation cycles of the pooling embryo transfer arm Two stimulation cycles for 49 women, three cycles for 30 women, four cycles for 15 women, five cycles for 8 women, six cycles for 6 women and seven cycles for 2 women were performed

**Figure 2:**
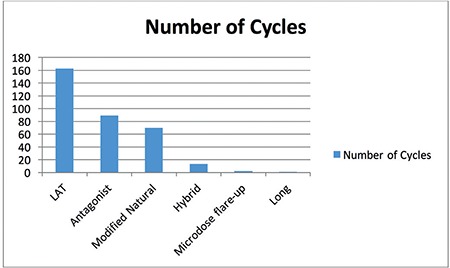
timulation protocols of the pooling embryo transfer arm The LAT protocol was used in 163 cycles, gonadotropin/antagonist protocol in 89 cycles, modified natural protocol in 70 cycles, hybrid protocol in 13 cycles, microdose flare-up protocol in 2 cycles and long protocol in 1 cycle *LAT: Letrozole/antagonist protocol*

One hundred ten patients for the pooling ET arm and 146 patients for the fresh ET arm were included in the study. In the fresh ET arm, one patient was excluded because of ectopic pregnancy. The demographic characteristics of both groups are displayed in Table 1. The age was similar in both treatment arms (p=0.31), but the antral follicle count (antral fdlicle count; p=0.001), total number of retrieved oocytes (p=0.001), total number of metaphase II (MII) oocytes (p=0.001), total gonadotropin dose (p=0.001), number of stimulation cycles (p=0.001), and cost of treatment (p=0.001) were significantly different. The day of ET was similar between the groups, but the number of transferred embryos was significantly higher in the pooling ET arm. The p values were 0.72 and 0.001, respectively (Table 1). In the pooling ET arm of the study, two stimulation cycles for 49 women, three cycles for 30 women, four cycles for 15 women, five cycles for 8 women, six cycles for 6 women, and seven cycles for 2 women were performed (Figure 1). In total, 338 stimulated cycles were performed in 110 women in the pooling ET arm. The protocols used in the stimulated cycles were as follows: the letrozole/antagonist protocol in 163 cycles, gonadotropin/antagonist protocol in 89 cycles, modified natural protocol in 70 cycles, hybrid protocol in 13 cycles, microdose flare-up protocol in 2 cycles, and long protocol in 1 cycle (Figure 2). 

In total, 495 oocytes were collected, 399 of which were MII oocytes. The mean oocyte number and MII oocyte number per one cycle were 1.59±0.69 and 1.3±0.63, respectively. In the fresh ET group, 145 stimulated cycles (flexible letrozole/antagonist) were applied to 145 women. At the end of these cycles, 332 oocytes were collected, 276 of which were MII oocytes. The mean oocyte number and MII oocyte number per one cycle were 2.29±0.75 and 1.9±0.75 (p<0.01), respectively. The pregnancy outcomes for both groups are displayed in Table 2. The positive test result and clinical pregnancy outcomes were similar between both arms (p=0.43 and 0.33, respectively). The implantation rate, live birth rate per patient, and live birth rate per transferred embryo were found to be significantly higher in the fresh ET arm (p=0.03, 0.04, and 0.003, respectively). The abortion rate was observed to be significantly higher in the pooling ET arm (p=0.04). Although binary comparisons revealed that the type of treatment and women’s age were effective variables in relation to live births, the total number of oocytes, total number of MII oocytes, number of transferred embryos, and day of ET were found to be ineffective variables (p=0.02, <0.001, 0.63, 0.95, 0.23, and 0.07, respectively) (Table 3). Logistic regression analyses were used to evaluate variables that affected the live birth rates, such as age, total number of oocytes, total number of MII oocytes, number of cycles, type of treatment, number of transferred embryos, and day of ET. None of these variables were identified as risk factors for live birth outcomes. Although the age and type of treatment were different in the binary comparisons, no difference was found in the logistic regression analyses. 

## Discussion

In the present study, we determined no favourable effect of pooled ET for live birth rates. Cobo et al.^(21)^ performed oocyte pooling on 724 poor responder patients in their study. Subsequently, they hey thawed all the oocytes and performed ICSI fertilisation. The live birth rate was higher in the pooling arm (36.4% vs 23.7%), and the authors reported similar outcomes for patients aged ≥40 years (15.8% vs 7.1%). Furthermore, another study suggested that oocyte or embryo accumulation might be useful for specific conditions, such as cystic fibrosis and X-linked microtubular myopathy^(22)^. Unfortunately, we could not determine any positive effect of embryo accumulation in our study. However, the patient selection criteria in this study were different from those used in previous works. Cobo et al.^(21)^ used the poor responder criteria described previously by Surrey and Schoolcraft^(23)^, whereas Chatziparasidou et al.^(22)^ included poor responders according to low AFC levels (AFC <7) and candidates for PGD. In contrast, we used the Bologna Criteria to identify poor responders^(14)^; this may have caused a lower follicle pool in our study group, and therefore, a worse oocyte quality than in other studies.

Aneuploidy is mostly related to the non-disjunction of chromosomes during the first meiotic division^(24)^. The presence of aneuploidy indicates poor quality oocytes. Maternal age is the most determinant factor regarding oocyte aneuploidy^(25)^. A low ovarian response during COS is related to the depletion of the follicular pool and displays ovarian aging^(24)^. Setti et al.^(26)^ found similar aneuploidy and abortion rates among 80 poor and normoresponder patients aged >35 years undergoing ICSI/PGD. However, several studies have associated higher abortion rates with a poor ovarian response^(24,27)^. In addition, this rising pattern has been documented for all age groups in which ≤3 oocytes have been retrieved. Sunkara et al.^(24)^ reported their abortion rate as 20% in women from whom 1-3 oocytes were retrieved, whereas the rate was 13.1% in the ≥15 oocytes group. There is a close relationship between the oocyte number and abortion rate. The foetal aneuploidy rate rises in accordance with a diminishing follicular pool^(27)^. Furthermore, the abortion rate increases with maternal aging^(24)^. In our study, the abortion rate for the pooling ET group was 52% (16/31), whereas it was 27% (13/49) for the fresh ET group. Although the total oocyte number was higher in the pooling ET arm (4.5±1.9 vs 2.2±0.75), 3±1.29 COS cycles on average were conducted to collect them. Nevertheless, the increasing number of total oocytes did not decrease the aneuploidy rates depending on maternal aging; hence, more aneuploidic embryos may have been generated in the pooling ET arm. Therefore, it is reasonable to judge the oocyte factor as relating to increased abortion rates. There is a positive correlation between the total number of retrieved oocytes and live birth rates in both poor and normoresponder women^(9,28)^. Schimberni et al.^(29)^ reported a 20.3% pregnancy rate per patient and 14.3% abortion rate between poor responder women aged 36-39 years with a single ET. Similar results were reported by Ata et al.^(30)^ in poor responder women aged 38.2±4.9 years when the researchers followed the natural cycle and picked up one oocyte. Branigan and Estes^(31)^ reported 27% implantation and 29.4% clinical pregnancy rates in poor responder women aged under 40 years, with 2.1 oocytes on average. The pregnancy rates in this study were given per ET. Cycle cancellations were not included for either treatment arm. Therefore, a higher pregnancy rate was obtained than reported in the literature^(32,33,34)^. In our study, the number of retrieved oocytes per one cycle was lower in the pooling ET arm compared with the fresh ET cycles (1.59±0.69 vs 2.29±0.75, p<0.01). Based on this finding, a worse follicular pool in the pooling ET arm compared with the fresh ET arm may have caused a worse oocyte quality. In our study, the positive pregnancy rates were 32% (35/110) in the pooling ET arm versus 37% (53/145) in the fresh ET arm (p=0.43). The abortion rates were 52% (16/31) and 27% (13/49), respectively (p=0.04). Fewer oocytes were collected per one cycle in the pooling ET arm compared with the fresh ET arm. This may be the reason for the clinically poor pregnancy outcomes in poor responders, and increased rates may be related to the embryo accumulation method.

### Study Limitations

Our study has some limitations. This was a retrospective study, and the groups were not randomised. There may have been bias in the patient selection. Women who were expected to exhibit lower IVF success after the clinical evaluation may have been moved to the pooling ET arm. This may have caused higher abortion and lower live birth rates in the pooling ET arm. The length of infertility did not exclude confounding factors causing infertility, such as additional disorders, because this was a retrospective study. Women were not randomised and offered alternative ET methods, such as pooling and fresh ET. 

## Conclusion

Although the pooling ET method may have a mild positive effect on clinical pregnancy rates, no additional effect was determined for live birth rates. In addition, the abortion rate increased in accordance with the clinical pregnancy rate; abortion may induce anxiety and depression in patients^(35)^. Further prospective, randomised, controlled studies are needed to investigate the effects of pooling ET on live births.

## Figures and Tables

**Table 1:**
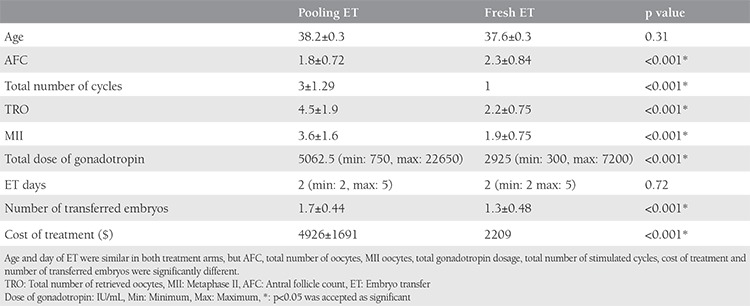
Demographic parameters of the patients

**Table 2:**
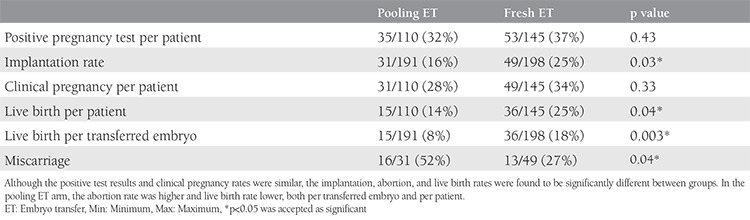
Pregnancy outcomes for both groups

**Table 3:**
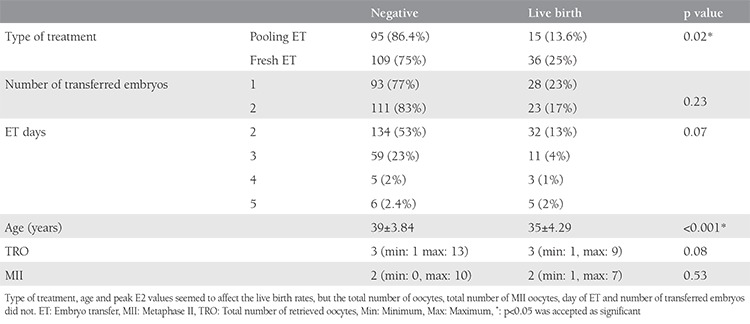
Variables affecting the live birth rates are displayed
